# Optimizing Protein Intake and Nitrogen Balance (OPINiB) in Adult Critically Ill Patients: A Study Protocol for a Randomized Controlled Trial

**DOI:** 10.2196/resprot.7100

**Published:** 2017-05-09

**Authors:** Matteo Danielis, Giulia Lorenzoni, Laura Cavaliere, Mariangela Ruffolo, Luca Peressoni, Amato De Monte, Rodolfo Muzzi, Fabio Beltrame, Dario Gregori

**Affiliations:** ^1^ Department of Anaesthesia and Intensive Care–Azienda Sanitaria Universitaria Integrata di Udine Udine Italy; ^2^ Unit of Biostatistics, Epidemiology, and Public Health Department of Cardiac, Thoracic, and Vascular Sciences University of Padova Padova Italy

**Keywords:** nitrogen balance, protein requirements, catabolism, mechanical ventilation, intensive care unit

## Abstract

**Background:**

Adequate nutrition of critically ill patients plays a key role in the modulation of metabolic response to stress.

**Objective:**

This paper presents the development of a protocol for a randomized controlled trial (RCT) aimed at comparing clinical outcomes of patients in the intensive care unit (ICU) administered with standard and protein-fortified diet. Together with the RCT study protocol, the results of the observational analysis conducted to assess the feasibility of the RCT are presented.

**Methods:**

An RCT on adult patients admitted to ICU and undergoing mechanical ventilation in the absence of renal or hepatic failure will be conducted. Patients enrolled will be randomized with an allocation rate of 1:1 at standard diet versus protein-fortified diet. The estimated sample size is 19 per arm, for a total of 38 patients to be randomized.

**Results:**

Enrollment began in January 2017. In the feasibility study, 14 patients were enrolled. Protein administration increased significantly (*P*<.001) over time but was significantly lower compared to that recommended (*P*<.001). Blood urea nitrogen significantly increased (*P*<.03) over the period of observation. Such increased catabolism resulted in negative cumulative nitrogen balance (NB) in all patients, and some patients presented with a more negative NB compared to the others.

**Conclusions:**

Results of the feasibility study clearly confirmed that protein provision in ICU patients is below that recommended and that this results in impaired NB. The emerging of an interindividual variability in NB will be further analyzed in the RCT.

**Trial Registration:**

ClinicalTrials.gov NCT02990065; https://clinicaltrials.gov/ct2/show/NCT02990065 (Archived by WebCite at http://www.webcitation.org/6prsqZdRM).

## Introduction

In recent years, several studies have focused on the molecular and biological effects of nutrients in maintaining homeostasis in patients admitted in intensive care unit (ICU), and ad hoc recommendations have been developed for the assessment and provision of nutritional support in adult critically ill patients [1,2]. In such patients, specific metabolic mechanisms are activated to face stresses related to critical conditions (eg, trauma and sepsis). These metabolic responses consist of changes in substrate utilization and substance synthesis rates, as well as catabolism and hypermetabolism, resulting in increased energy expenditure, hyperglycemia, loss of body mass, and eventually psychological and behavioral problems [3]. Given such framework, monitoring the metabolic response is crucial in the management of ICU patients. However, this represents a major clinical challenge since it is generally assessed indirectly using nonspecific clinical and biochemical markers such as secondary infections, muscle atrophy and weakness, respiratory insufficiency, delayed wound healing, and incidence of secondary complications indicating prolonged catabolism [4]. Adequate nutrition plays a key role in the modulation of metabolic response to stress, contributing to the prevention of oxidative cellular injury and positively modulating immune responses.

All patients admitted in ICU require a full nutritional assessment for determining both energy and protein requirements to prevent malnutrition. According to international guidelines [2], the best approach is to reach the energy goal by indirect calorimetry (IC) when available. In the absence of IC, a predictive formula or simple weight-based equation (25-30 kcal/kg/day) may be used to determine energy requirements [2]. Although no consensus has been reached about the most accurate formula to be used in ICU, the Harris-Benedict equation (HBE) is the most often employed to estimate the resting energy expenditure (REE) in ICU patients [5]. However, these equations suffer from several limitations, including poor accuracy. Their poor accuracy is related to the fact that the variables affecting energy expenditure in critically ill patients (eg, weight, medications, treatments, and body temperature) are sensitive to changes over time. For mechanically ventilated patients in ICU, the main factors influencing REE have been found to be weight, height, body temperature, and minute ventilation [6].

In the critical setting, protein is the macronutrient most often lacking in such patients, and its supplementation is likely to result in beneficial effects [7]. Disorders of protein metabolism are documented as physiologic responses to stressful events and are reflected by important nitrogen loss and muscle wasting, which are proportional to the severity of illness [8]. Current evidence supports the early administration of protein supplementation because a stressful event alters homeostatic balance, resulting in an increased protein catabolism [9]. Administering exogenous protein or amino acid is crucial to reduce the breakdown of endogenous proteins by providing an alternative source of amino acids for gluconeogenesis and protein synthesis. Protein-energy deficit is associated with an increased rate of infection, poor wound healing, reduced respiratory muscle mass, and delayed weaning from mechanical ventilation, resulting in increased length of stay in ICU and increased care costs [10]. A high protein intake, estimated using a weight-based equation (1.2-2 g/kg/day), is recommended during the ICU stay regardless of the simultaneous caloric intake [9,11].

This paper presents the development of a protocol for a randomized controlled trial (RCT) aimed at assessing changes of nitrogen balance (NB) in ICU patients administered with standard parenteral/enteral diet and protein-fortified parenteral/enteral diet. Together with the study protocol, results of the pilot study conducted to assess the feasibility of such a trial are also presented.

## Methods

### Randomized Controlled Trial

#### Study Design and Randomization

This study has been designed as a parallel arm RCT enrolling patients admitted at the Department of Anaesthesia and Intensive Care *–* Azienda Sanitaria Universitaria Integrata di Udine (Italy). Medical and surgical adult patients admitted to ICU undergoing mechanical ventilation at the time of admission or in the first 12 hours will be enrolled. See Textbox 1 for selection criteria.

**Selection criteria.**

Inclusion criteria:

- Aged 18 years and older
- Receiving parenteral nutrition or enteral nutrition
- Having an indwelling catheter
- Undergoing mechanical ventilation

Exclusion criteria:

- Current or 6-month past history of gastrointestinal bleeding
- body mass index <18.5 kg/m^2^ or ≥ 30 kg/m^2^
- Terminal illness
- Pregnancy
- Acute renal failure defined using Kidney Disease Improving Global Outcomes (KDIGO) Clinical Practice Guidelines [12]: patients with a KDIGO stage 2 (serum creatinine 2-2.9 times baseline or urine output <0.5 mL/kg/h for 12 hours) and 3 (increase in serum creatinine ≥4 mg/dL, anuria for more than 12 hours, or starting of renal replacement therapy)
- Hepatic failure

**Figure 1:**
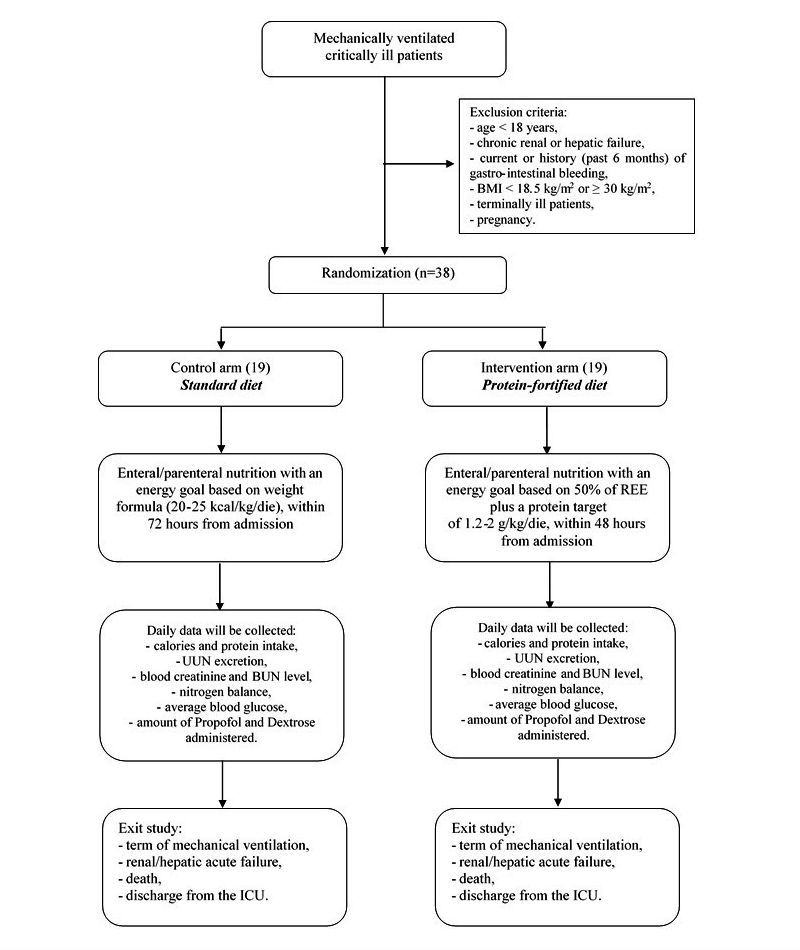
Flowchart of the study protocol.

Patients enrolled will be randomized to standard nutritional care (standard diet) to meet daily patient caloric requirement (control group) or appropriate amount of protein feeding (protein-fortified diet) to meet daily patient protein requirement (intervention group). Random allocation will be performed using a computer-generated algorithm (with an allocation rate of 1:1). Subjects enrolled will remain in the study until they are no longer mechanically ventilated. Participants will be blinded to treatment allocation. The flowchart of the study protocol is shown in Figure 1.

#### Treatment Arms

The standard diet consists of an energy goal based on weight formula (20-25 kcal/kg/day). According to the ICU nutritional protocol, enteral nutrition (EN) will be started at an initial rate of 10 mL/h and increased by 20mL/h every 12 hours in the absence of significant gastric residuals (<250 mL), with the aim of reaching the energy goal within 72 hours of admission. The EN formulae used are standard (1-1.5 kcal/mL, 40 g/L protein). If EN is not tolerated or not indicated, supplemental parenteral nutrition (PN) will be used to make up the energy shortfall. The PN formula used is standard (1000 kcal/L, 37 g/L protein).

The protein-fortified diet consists of an energy goal based on REE and a protein target based on the most recent literature recommendations (1.2-2 g/kg/day) [2]. Daily caloric requirement and subsequent protein content for patients enrolled in the intervention group will be calculated using formulae reported in Multimedia Appendix 1 [10,13,14]. For each patient, it will be calculated based on the REE and the daily protein requirement (1.2-2 g/kg/day of body weight registered at time of admission) and the corresponding caloric intake (1 g=4 kcal). Daily total caloric intake will be represented by 50% protein (1 g=4 kcal) and another 50% nonprotein (ie, fat and carbohydrates).

For the intervention group, EN will be started at an initial rate of 10 mL/h and increased by 20 mL/h every 8 hours in the absence of significant gastric residuals (<250 mL) with the aim of reaching the energy goal within 48 hours of admission. The EN formulae used are high in protein (1-1.5 kcal/mL, 74 g/L protein). If EN is not tolerated or not indicated, supplemental PN is used to make up the energy shortfall. The PN formula used will be arranged by the hospital pharmacy to meet the energy targets set for the intervention group.

For patients enrolled in the control group and the intervention group, gastric residuals will be checked 4 times per day and electrolytes will be closely monitored and replaced. Adequacy of nutritional support will be determined daily by measuring NB.

#### Data Collection

Age (years), gender, weight (kg), height (cm), body mass index (BMI) (kg/m^2^), main diagnosis, clinical history, and illness severity assessed using the Acute Physiology and Chronic Health Evaluation (APACHE) II score [15] will be collected at time of admission in ICU (baseline assessment). The Simplified Acute Physiology Score (SAPS) II for mortality prediction in ICU [16] will also be calculated.

The following parameters will be recorded at midnight of each day: caloric intake, protein intake, measurement of 24-hour urine urea nitrogen (UUN) excretion, blood creatinine, NB, blood urea nitrogen (BUN) level, average blood glucose, amount of propofol administered (mL/day), amount of dextrose administered (mL/day).

A standard formula will be used for NB calculation [17,18]: total protein intake (g)/6.25 –(UUN + 4 g), where 6.25 = 6.25 g of protein per gram of nitrogen, UUN = grams of nitrogen excreted in the urine over 24-hour period of time, and 4 = 4 g of nitrogen lost each day as insensible losses via the skin and gastrointestinal tract. Additionally, for each patient, the duration of mechanical ventilation and any infection or skin alteration discovered during the study period will be reported.

#### Sample Size

Considering NB as primary outcome, a sample size estimation has been performed considering a *t* test difference in means between the 2 groups (standard and protein-fortified diet). For a specified alpha of .025 and a power of 0.90, which aimed at detecting a difference of at least –6 g of NB between the 2 groups (assuming an equal standard deviation in the 2 groups of 5 g), the estimated sample size is 19 per arm for a total of 38 patients to be randomized. The sample size has been estimated using the sample size package in R software (The R Foundation) [19].

Data will be entered and managed using REDCap (Research Electronic Data Capture), a Web-based application for managing databases hosted at the Department of Cardiac Thoracic and Vascular Sciences, University of Padova (Italy).

#### Statistical Analysis Plan

The primary endpoint will be analyzed for the intention-to-treat (ITT) population. After having reached the sample size foreseen, a *t* test with an alpha level equal to .025 will be performed to assess the statistical differences in NB between protein-fortified diet and standard diet arms. As secondary endpoint, the hospital mortality rate will be considered. On the ITT population, the secondary endpoint will be evaluated by testing the difference between the 2 groups in a logistic model framework (alpha=.025).

The study was approved by the regional ethics committee of Friuli Venezia Giulia, Italy (CEUR-2016-Sper-066-ASUIUD). Each patient or legally authorized representative must provide written informed consent for the study procedures.

### Feasibility Study

#### Study Design

An observational analysis was conducted to assess the feasibility of the trial. This observational study enrolled patients admitted to the ICU *of* the Department of Anaesthesia and Intensive Care *–* Azienda Sanitaria Universitaria Integrata di Udine (Italy). The study was undertaken in a group of mixed medical, surgical, and trauma patients undergoing mechanical ventilation. Exclusion criteria were age (less than 18 years), chronic renal (identified using KDIGO recommendations) or hepatic failure, current or 6-month past history of gastrointestinal bleeding, body mass index <18.5 kg/m^2^or ≥30 kg/m^2^, terminal illness, and pregnancy. This study aimed at investigating the level of energy intake (kcal and protein) and nitrogen excretion in an ICU population receiving standard diet.

#### Treatment

Our nutritional approach was led by current guidelines that recommend providing 25-30 kcal/kg/day. Both EN and PN were used to achieve energy goals. EN formula was the Nutrison standard (1000 kcal and 40 g of protein per 1000 mL); an all-in-one solution containing 1000 kcal and 47 g of protein per 1000 mL was used for PN.

#### Data Collection

Using REDCap, the following patient information were collected: admission diagnosis, comorbidities, age, sex, BMI, APACHE II score, SAPS II score (at baseline), type and amount of nutrition received (both caloric and protein intake), amount of propofol and dextrose (mL/day) administered, and blood chemistry (urea nitrogen level, creatinine, glucose) (at midnight of each day). A daily 24-hour urine collection was conducted in all patients and NB was calculated. For each patient, any infection or skin alteration occurring during the study period was reported. Daily data were collected until the end of mechanical ventilation or until renal/hepatic acute failure, death, or discharge from the ICU.

#### Data Analysis

Continuous variables were reported as median (I quartile and III quartile), and discrete parameters were reported as absolute value (percentage). The distribution of the quantitative variables was summarized using simple barplots for trend. To assess if the series has an increasing or decreasing trend, a nonparametric Spearman test was performed between the observations and time. To calculate trends, means of patient measures of interest were considered. A *t* test was carried out to test the significance of mean difference from the amount of protein actually administered and that recommended by international guidelines. All analyses were performed using R software (The R Foundation) [19].

## Results

### Randomized Controlled Trial

Enrollment in the study began in January 2017. Data collection is expected to be conducted until April 2017. Data analysis will start once the data collection is completed and the database is locked.

### Feasibility Study

Sample characteristics are summarized in Table 1. A total of 14 patients were enrolled, with a median age of 48 years. Median weight was 83 kg, and median BMI was 25.5 kg/m^2^. For about a half of the patients (6/14, 43%) the main diagnosis reported at time of admission was trauma (especially multiple trauma). Of the 14 patients evaluated, 8 (57%) completed the observation until the end of mechanical ventilation.

**Figure 2:**
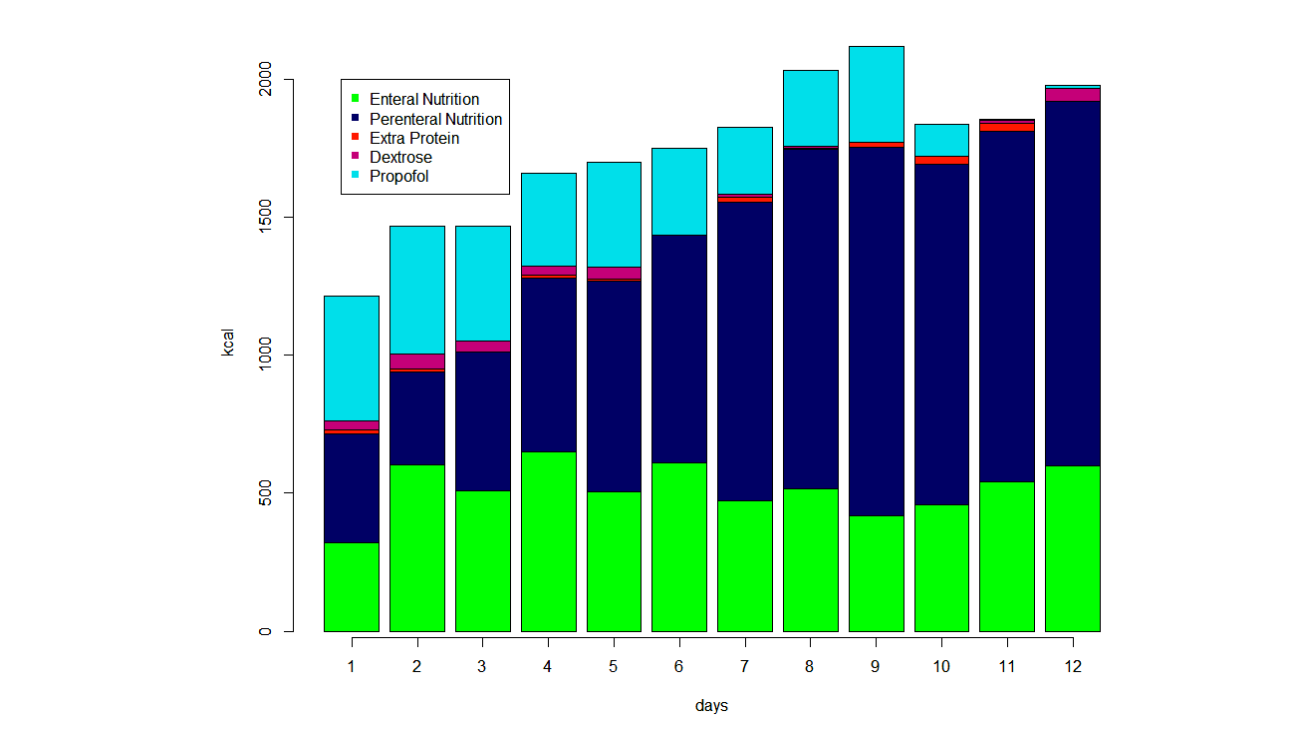
Composition of the calories administered.

**Figure 3:**
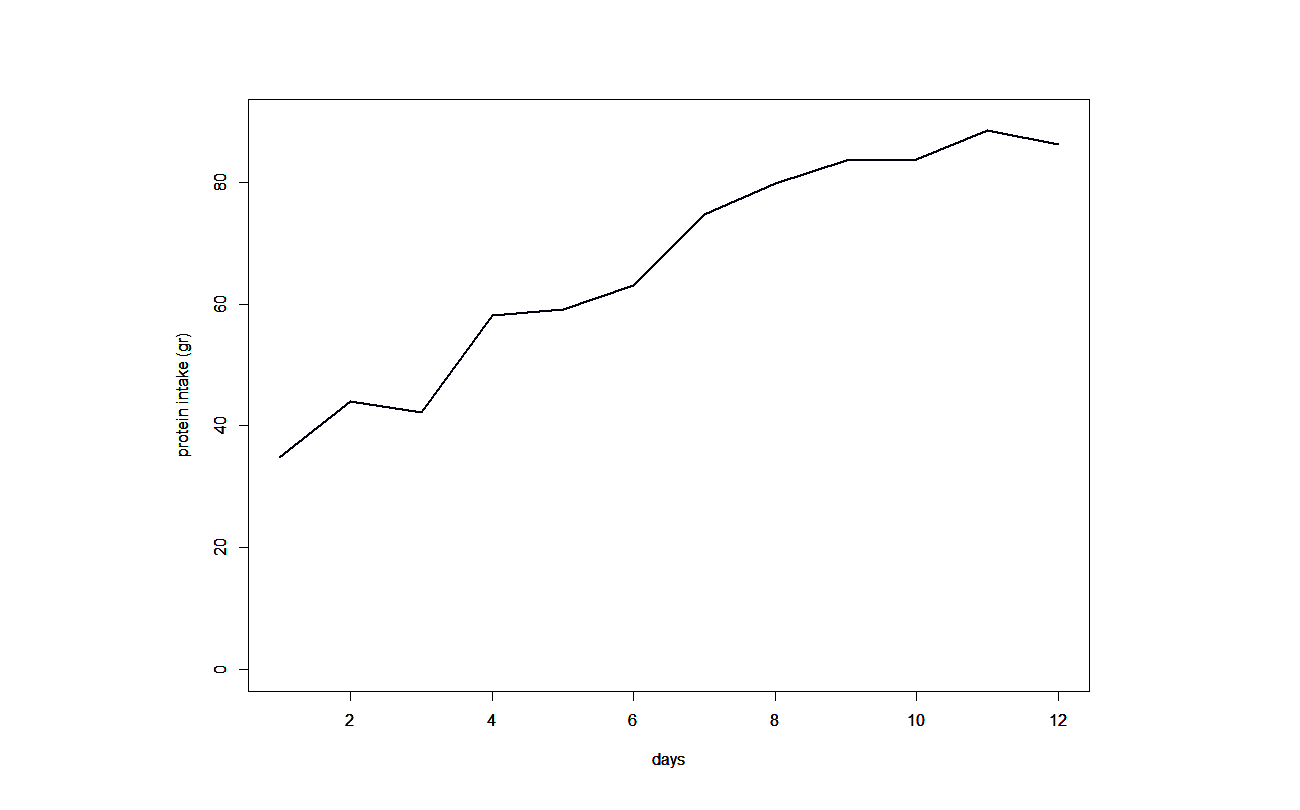
Protein intake trend.

Figure 2 shows the contribution of EN, PN, extra proteins (represented by albumin), and propofol and dextrose infusions to the mean energy (kcal) intake over the feasibility study period. During the first 3 days of observation, propofol contributed to about half of the daily caloric intake. Additionally, calories introduced by PN increased over the days, while calories provided by EN remained stable. Overall, caloric intake increased over time. Along with caloric intake, protein administration also increased significantly (*P*<.001) over the period of observation (Figure 3).

**Figure 4:**
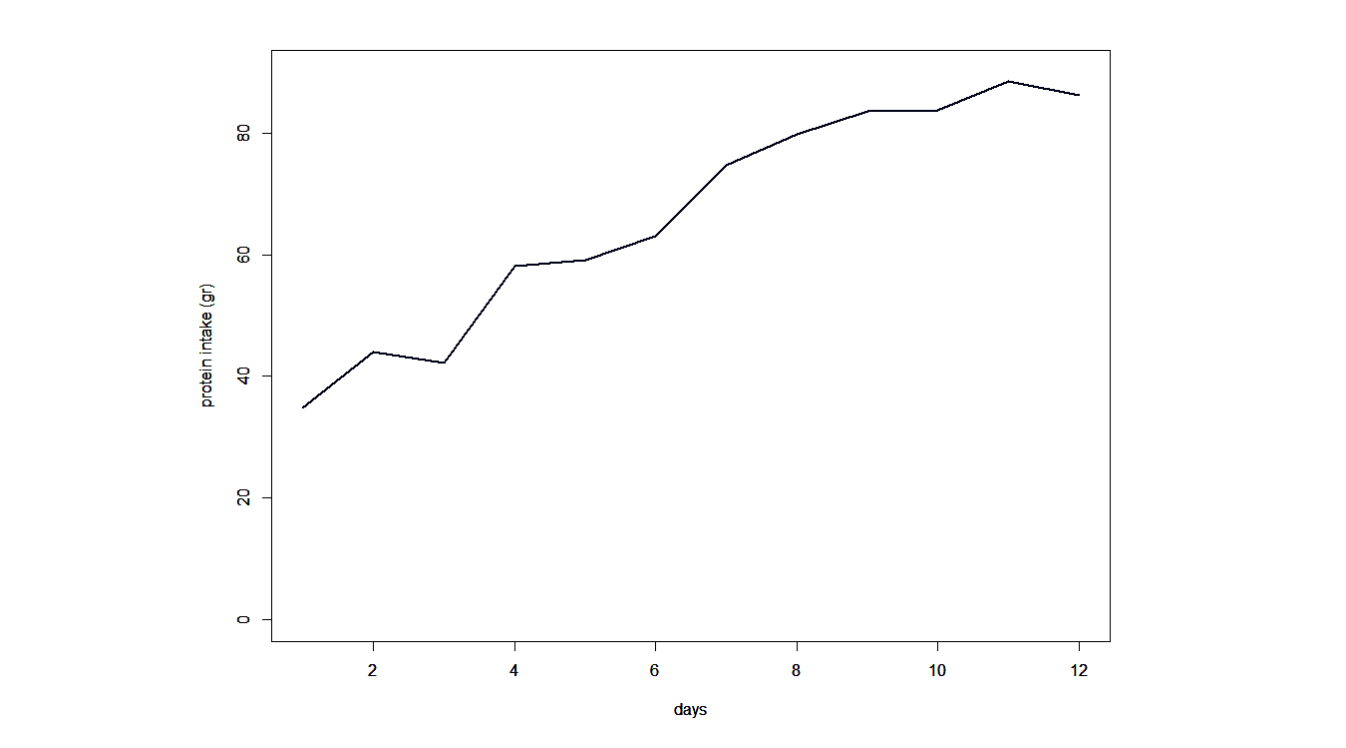
Blood urea nitrogen trend.

**Figure 5:**
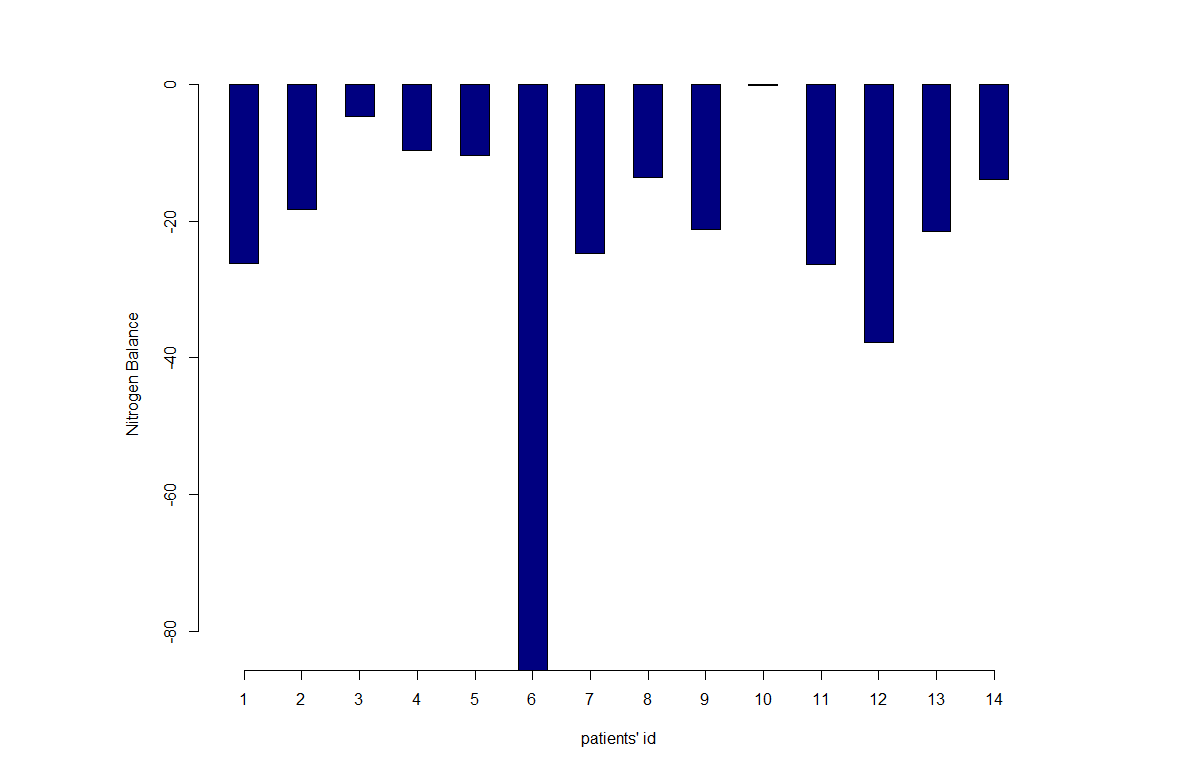
Cumulative nitrogen balance during the first 72 hours.

Despite the increased provision of protein during hospitalization, the BUN significantly increased (*P*=.03) over the period of observation (Figure 4), thus indicating an increased catabolism in patients enrolled (since urea represents the waste product from protein metabolism). Increased catabolism resulted in negative NB, as shown in Figure 5. All patients had a negative cumulative NB in the first 72 hours of observation, and some patients presented with a markedly more negative NB compared to the others.

**Figure 6:**
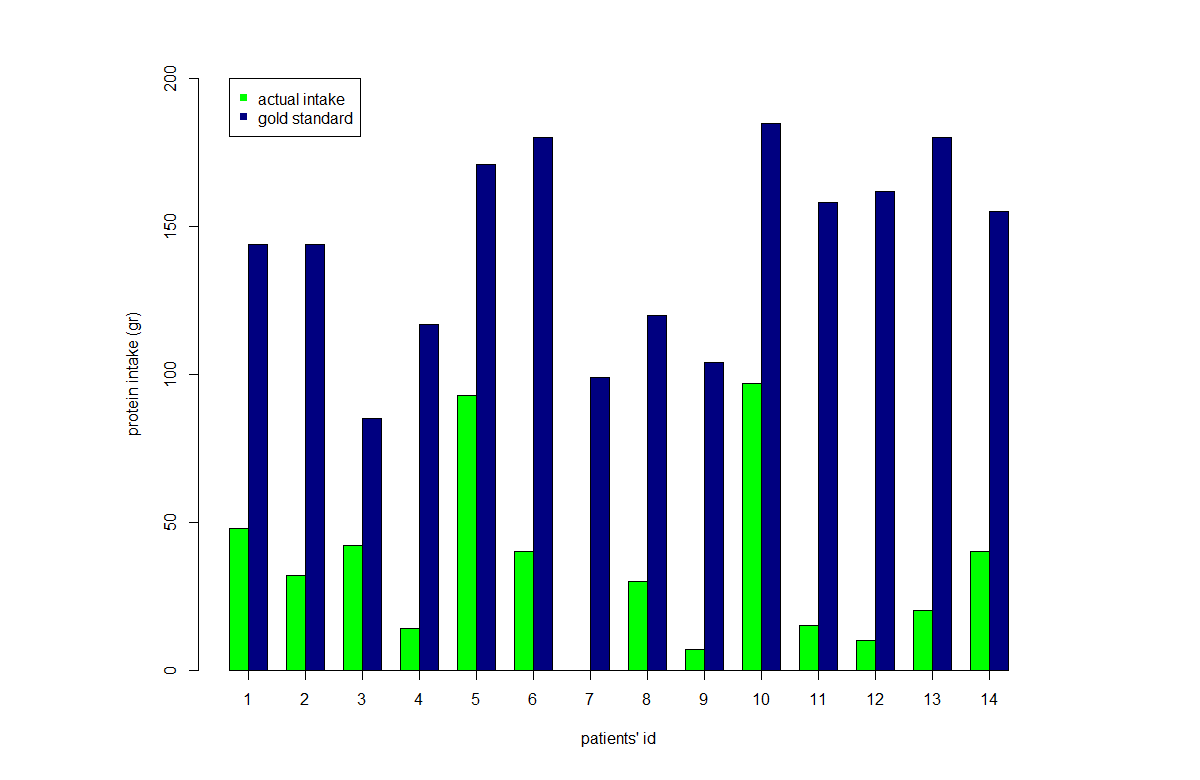
Protein intake administered versus ideal protein target in the first 24 hours.

Finally, Figure 6 reports the comparison between patients’ actual protein intake and that recommended by international guidelines (considering a value of 1.8 g/kg/day in the range between 1.2 and 2 g/kg/day). Clearly, the amount of protein actually administered is significantly lower compared to that recommended (*P*<.0001) (for patient 7, PN/EN was not administered in the first 24 hours due to medical procedures, resulting in fasting). The median difference between actual and recommend protein intake was found to be –360 g/kg (range –638 to 52).

**Table 1:** Characteristics of the patients enrolled in the feasibility study.

Characteristics			Number
Age (years), median (I and III quartile)			48 (43-55)
**Sex, n (%)**			
	Male		8 (57)
	Female		6 (46)
Weight (kg), median (I and III quartile)			83 (66-94)
BMI^a^(kg/m^2^), median (I and III quartile)			25.5 (24.2-27)
APACHE^b^II score, median (I and III quartile)			10.5 (9-16)
SAPS^c^II score, median (I and III quartile)			29 (24-36)
Observation period (days), median (I and III quartile)			8 (6-12)
**Admission diagnosis, n (%)**			
	Organ failure		4 (29)
		Respiratory failure	3 (75)
		Sepsis and infection	1 (25)
	Trauma		6 (43)
		Multiple trauma	5 (83)
		Spinal trauma	1 (17)
	Cerebrovascular disease		4 (29)
		Cerebral hemorrhage	2 (50)
		Coma	2 (50)
**Comorbidities, n (%)**			
	Cerebrovascular accident		3 (21)
	Chronic obstructive pulmonary disease		1 (7)
	Diabetes		2 (14)
	Hypertension		3 (21)
	Smoking		2 (14)
	Anxiety/depression		2 (14)
	Neoplasia		1 (7)
**Exit study, n (%)**			
	Transfer to another hospital		2 (14)
	Term of mechanical/spontaneous breathing		8 (57)
	Acute renal or hepatic failure		3 (21)
	Death		1 (7)

^a^BMI: body mass index.

^b^APACHE: Acute Physiology and Chronic Health Evaluation.

^c^SAPS: Simplified Acute Physiology Score.

## Discussion

### Principal Findings

In recent years, several studies have focused on the importance of an adequate caloric intake in critically ill subjects. However, specific evidence about protein intake (adequate amount to be administered, adverse outcomes associated with inadequate protein intake) is sparse, even though several studies have demonstrated that in critically ill patients protein breakdown is greatly increased [3,11,20]. Adequate provision of protein represents a relevant issue in patients admitted in ICU, because pathological conditions such as surgical procedures and infection diseases result in increased protein requirements [11,21]. A high protein intake is recommended during the early phase of the ICU stay due to increased catabolism [9]. Allingstrup et al [22] observed a lower mortality in patients receiving a mean of 1.46 (SD 0.29) g/kg/day than those receiving 0.79 (SD 0.29) or 1.06 (SD 0.29) g/kg/day. This suggested that an optimal protein provision may result in better clinical outcomes compared to an optimal caloric provision alone in critically ill patients [21].

Considering this framework, the main issue in the nutritional management of ICU patients is represented not only by the identification of the most adequate daily caloric intake but also by the identification of the optimal contribution of each nutrient to the daily caloric intake, focusing particularly on protein content. The aim of our study protocol is to provide evidence about the effectiveness of a protein-fortified diet in patients admitted to ICU.

To assess the feasibility of this study protocol, a pilot study was conducted. Consistent with previous research [3,11], our feasibility study confirmed that critically ill patients had a severe protein catabolism that resulted in a strong negative NB. Literature has suggested that this situation may result in prolonged ventilator dependence and increased risk of brain dysfunction, neuromuscular weakness, metabolic changes, muscle wasting, malnutrition, skin breakdown, and symptoms distress like pain, anxiety, and depression [23].

Although the NB was negative in all patients, we observed that cumulative NB in the first 72 hours was different among subjects observed. This is probably due to an interindividual variability in the lean body mass that results in a different amount of protein metabolism waste. Beyond its action on protein metabolism, dietary protein intake affects body composition [18]. This interindividual variability might be considered in the estimation of protein intake. However, while a high protein intake is able to preserve lean mass, especially during the early or most catabolic phases of illness, the specific goal of protein requirement to minimize the loss of lean mass is not yet clear [21,24]. Additionally, together with the marked negative NB, actual protein intake was found to be dramatically lower than that recommended by international guidelines for patients undergoing mechanical ventilation.

Weight-based equations (1.2-2 g/kg/day) may be used to monitor adequacy of protein provision [2,25], but in adult critically ill patients admitted to ICU, it is difficult to achieve this target. This is because standard nutritional formulations are rich in lipid and carbohydrate calories, but the protein content is inadequate. Furthermore, our findings showed that the contribution of EN to caloric intake remained stable over the period of observation. This may be attributable to feeding intolerance and high gastric residual volumes or to interruptions for medical procedures (eg, bronchoscopy, computed tomography, as with patient 7). If EN is insufficient, supplemental PN should be considered [9]. However, even if PN administration progressively increased during the period of observation, the composition of both PN or EN did not allow the achievement of protein optimal requirements.

### Conclusions

Even though several studies have shown that the provision of an appropriate protein intake may reduce net muscle catabolism, to our knowledge this is the first study protocol aimed at comparing clinical outcomes of standard and protein-fortified diet in an ICU population. Results of the feasibility study clearly confirmed that protein provision in ICU patients is below that recommended in international guidelines and that this results in impaired NB. Moreover, it provided evidence that protein catabolism is different among patients, probably due to differences in body composition (eg, lean body mass). Such interindividual variability will be further analyzed in the trial to understand if and how it may be considered in the titration of protein intake.

## Appendix

Multimedia Appendix 1 Energy and protein intake calculation for intervention group only.

Multimedia Appendix 2 Peer review and approval from Etichs Committee.

## Abbreviations

APACHE: Acute Physiology and Chronic Health Evaluation
BMI: body mass index
BUN: blood urea nitrogen
EN: enteral nutrition
HBE: Harris-Benedict equation
IC: indirect calorimetry
ICU: intensive care unit
ITT: intention-to-treat
KDIGO: Kidney Disease Improving Global Outcomes
NB: nitrogen balance
PN: parenteral nutrition
RCT: randomized controlled trial
REDCap: Research Electronic Data Capture
REE: resting energy expenditure
SAPS: Simplified Acute Physiology Score
UUN: urine urea nitrogen
