# Supplementary material for: Optimizing Protein Intake and Nitrogen Balance (OPINiB) in Adult Critically Ill Patients: A Study Protocol for a Randomized Controlled Trial
Source: JMIR Res Protoc. 2017 May 9;6(5):e78. doi: 10.2196/resprot.7100 (PMC5442349; doi:10.2196/resprot.7100)
Supplement: Multimedia Appendix 1 [file resprot_v6i5e78_app1.pdf]

1) REE (resting energy expenditure kcal/day)			
Patient <60 years		Patient > 60 years	
8 x weight (kg) + 14 x height (cm) + 32 x minute ventilation (L/min) + 94 x temperature (degrees °C) – 4834		(0.85 x HBE) + (175 x temperature degrees °C) + (33 x minute ventilation (L/min)) – 6433<br>HBE (Harris Benedict equation):<br>Men = 66.4730 + (13.7516 x weight) + (5.0033 x height) – (6.7550 x age)<br>Women = 655.0955 + (9.5634 x weight) + (1.8496 x height) – (4.6756 x age)	
TOT (Kcal)		TOT (Kcal)	
2) Protein intake			
1.2 g x weight (kg)	1.5 g x weight (kg)	1.8 g x weight (kg)	2 g x weight (kg)
TOT (Kcal)	TOT (Kcal)	TOT (Kcal)	TOT (Kcal)
TOTAL ENERGY COUNTING (kcal) = REE x 0.5 + Protein Intake			
