# Supplementary material for: Optimizing Protein Intake and Nitrogen Balance (OPINiB) in Adult Critically Ill Patients: A Study Protocol for a Randomized Controlled Trial
Source: JMIR Res Protoc. 2017 May 9;6(5):e78. doi: 10.2196/resprot.7100 (PMC5442349; doi:10.2196/resprot.7100)
Supplement: Multimedia Appendix 2 [file resprot_v6i5e78_app2.pdf]

REGIONE AUTONOMA FRIULI VENEZIA GIULIA
ente per la gestione accentrata dei servizi condivisi
EGAS

SEDE LEGALE: Udine - Via Pozzuolo 330
Centralino: +39 0432 554160 – Fax: +39 0432 306241
C.F./P.IVA 02801630308
e mail: segreteria@egas.sanita.fvg.it - PEC:egas.protgen@certsanita.fvg.it

COMITATO ETICO UNICO REGIONALE
sede operativa CENTRO di RIFERIMENTO ONCOLOGICO
Istituto di ricovero e cura a carattere scientifico
Via Franco Gallini, 2 – 33081 AVIANO (PN) tel. 0434 659282

Prot. n. 25742
Presidente CEUR: dr. Paolo Rossi
Responsabile Segreteria tecnico-scientifica:
dott. Paolo De Paoli
Referente della pratica: Caterina Comand
Tel. +39 0432 552206
Fax +39 0432 306241
e-mail: caterina.comand@egas.sanita.fvg.it

Egregio
Dr. Amato De Monte
Dip. Anestesia e Rianimazione
ASUI di Udine
Demonte.amato@aoud.sanita.fvg.it

Gent.ma Sig.ra Elda Cameranesi
Direttore SOC Affari Generali ASUI di Udine

Udine, lì 9 NOV. 2016

OGGETTO: (Odg 3.3 seduta 08.11.2016) –Studio sperimentale randomizzato controllato monocentrico no profit intitolato "**O**ptimizing **p**rotein **i**ntake and **ni**trogen **b**alance in adult critically ill patients: a randomized controlled trial" "Ottimizzare l'assunzione di proteine e il bilancio azotato nei pazienti adulti in condizioni critiche: studio randomizzato controllato" – **Studio OPINiB – Promotore: Dipartimento Anestesia e Rianimazione – SOC Anestesia e Rianimazione 1-2 A.S.U.I. Udine – Sperimentatore responsabile per le SOC Anestesia e Rianimazione 1 e 2 A.S.U.I. Udine: Dott. Amato De Monte – Direttore di Dipartimento.**

Con riferimento alla precedente nota prot. n. 24155 del 19.10.2016 si comunica che questo Comitato nella seduta del 08.11.2016, ha preso atto dei chiarimenti inviati dal Direttore della SOC Affari Generali dell'ASUI di Udine, tramite e-mail in data 24.10.2016 e successive integrazioni del 02.11.2016, nelle quali viene confermata la sussistenza di idonea copertura assicurativa aziendale per lo studio in oggetto. Il Comitato ha quindi sciolto le riserve in relazione agli aspetti assicurativi.

Per quanto attiene le modifiche richieste al protocollo, il Comitato ritiene che queste siano state interamente accolte:

- **Protocollo v. 3**

Il Comitato esprime pertanto **Parere favorevole** per lo studio in oggetto.

Parere CEUR-2016-Sper-066-ASUIUD

Il Comitato Etico Unico Regionale, istituito ai sensi del Decreto 8 febbraio 2013 e Legge Regionale n. 33 del 29.12.2015, con DGR 22 gennaio 2016 n. 73, Decreto n. 414/SPS del 31.03.2016, e Decreto 694/SPS del 31.05.2016, opera in osservanza a quanto previsto dal DM 15 luglio 1997, dalla Circolare n. 15 del 15 ottobre 2000, dal D. Lgs n. 211 del 24 giugno 2003, e nel rispetto delle norme di Buona Pratica Clinica (GCP-ICP).

Le decisioni sono assunte a maggioranza assoluta dei presenti aventi diritto al voto. I componenti del Comitato si sono astenuti dal pronunciarsi su quelle sperimentazioni per le quali possa sussistere un conflitto di interesse di tipo diretto o indiretto.

Questo Comitato Etico dovrà essere informato della data di inizio e di conclusione dello studio, dell'eventuale sua sospensione od interruzione, per qualsiasi causa.

Si rammenta che la presente lettera non costituisce autorizzazione all'avvio dello Studio, presso la Struttura Operativa in indirizzo, e che detta autorizzazione dovrà essere formalizzata con successivo decreto della Direzione aziendale.

Distinti saluti

Il Presidente Comitato Etico Unico Regionale
Dr. Paolo ROSSI

All.: Foglio firme dd 08.11.2016

COMITATO ETICO UNICO REGIONALE
(Deliberazione di Giunta Regionale n. 72 del 22.01.2016; Decreto della Direzione Centrale Salute integrazione sociosanitaria politiche sociali e famiglia n. 414/SPS del 31.03.2016; Decreto del Commissario Straordinario Egas n. 31 del 26.04.2016)

Seduta del 8 novembre 2016

	**firma PRESENTI**
Clinico 1: **dott. Paolo ROSSI – Presidente C.E.U.R.**<br>Medico internista – Dip. Medicina Interna, AOU di Udine	
Clinico 2: **dott. Claudio FRESCO**<br>Cardiologo - SOC Cardiologia, AOU di Udine	
Clinico 3: **prof. Francesco ZAJA**<br>Ematologo - SOC Clinica Ematologica, AOU di Udine	
Clinico 4: **prof. Paolo MANGANOTTI**<br>Neurologo – AOU di Trieste	
Clinico 5: **dott.ssa Alessandra BEARZ**<br>Oncologo - IRCCS CRO di Aviano	
Clinico 6: **dott. Cosimo Stanislao SACCO**<br>Oncologo – Dip. Oncologia, AOU di Udine	
Clinico 7: **dott. Fabio FISCHETTI**<br>Reumatologo – AOU di Trieste	
Pediatra 1: **dott.ssa Grazia DI LEO**<br>IRCCS BURLO di Trieste	
Pediatra 2: **dott. Andrea TADDIO**<br>IRCCS BURLO di Trieste	
Statistico 1: **dott. Jerry POLESEL**<br>IRCCS CRO di Aviano	
Statistico 2: **prof.ssa Miriam ISOLA**<br>Università degli Studi di Udine	
Farmacologo: **prof. Massimo BARALDO**<br>SOC Istituto di Farmacologia Clinica, AOU di Udine	
Bioeticista: **mons. Bruno PIGHIN**	
Genetista: **prof. Mauro GIACCA**<br>Ingegneria Genetica e Medicina Molecolare, ICGEB Trieste	Assente
Farmacista S.S.R: **dott. Paolo SCHINCARIOL**<br>S.C. Farmacia, AOU di Trieste	Assente
Medico di Medicina Generale: **dott. Luciano PRELLI**<br>Rappresentante Medicina Territoriale	
Pediatra di Libera Scelta: **dott. Paolo PECILE**<br>Rappresentante Medicina Territoriale	

COMITATO ETICO UNICO REGIONALE
(Deliberazione di Giunta Regionale n. 72 del 22.01.2016; Decreto della Direzione Centrale Salute integrazione sociosanitaria politiche sociali e famiglia n. 414/SPS del 31.03.2016; Decreto del Commissario Straordinario Egas n. 31 del 26.04.2016)

Seduta del 8 novembre 2016

	**firma PRESENTI**
Rappresentante Area Professioni Sanitarie: **Ilario GUARDINI** AOU di Udine	[signature]
Medico legale: **dott.ssa Barbara POLO GRILLO**	[signature]
Esperto in dispositivi medici: **dott. Paolo GIRIBONA**	[signature]
Rappresentate del Volontariato **Renza ZANON**	[signature]
**COMPONENTE di SEDE** **ex-officio *o sostituto:***	
ASUI di Trieste: **dott. ssa Lucia PELUSI** sostituto	[signature]
AAS n. 2 "Bassa Friulana-Isontina": **dott.ssa Claudia GIULIANI** sostituto	[signature]
AAS n. 3 "Alto Friuli – Collinare – Medio Friuli": **dott.ssa Paola ZULIANI** sostituto	Zuliani Paola
AAS n. 5 "Friuli Occidentale": : **dott.ssa Paola TOSCANI** Direzione sanitaria - Componente ex officio	[signature]
ASUI di Udine: **dott.ssa Rosanna QUATTRIN** sostituto	[signature]
IRCCS CRO di Aviano: **dott. Paolo DE PAOLI** Direttore Scientifico - Componente ex officio	[signature]
IRCCS BURLO di Trieste: **dott. Luca RONFANI** sostituto	Assente
**COMPONENTI IN RELAZIONE ALLO STUDIO CLINICO**	
Nutrizionista esperto in nutrizione umana: **prof. Gianni BIOLO** Medicina Interna – AOU di Trieste	Assente
Specialista in terapia intensiva: **dott.ssa Adriana DI SILVESTRE** SOC Anestesia e Rianimazione, AOU di Udine	[signature]
Ingegnere Clinico: **ing. Massimo D'ANTONI** SOC Ingegneria Clinica, AOU di Udine	[signature]
